# Climate‐Driven Advanced Machine Learning Approach for Dengue Incidence Forecasting in Bangladesh

**DOI:** 10.1002/hsr2.72207

**Published:** 2026-03-27

**Authors:** Omar Faruk

**Affiliations:** ^1^ Department of Civil Engineering Dhaka International University Dhaka Bangladesh

**Keywords:** Bangladesh epidemiology, dengue incidence forecasting, LSTM, machine learning, meteorological drivers, NBR, SARIMAX, XGBoost

## Abstract

**Background and Aims:**

Dengue fever has become a significant and an increasing public health menace in Bangladesh. Despite the abundance of research on the dengue outbreaks, the majority of the studies are limited by the short‐term time frame of research, a narrow scope, or to one modeling methodology. Thus, a gap in the existing knowledge on long‐term transmission processes, climatic predictors, and most importantly, the comparative validity of rival forecasting frameworks of dengue in Bangladesh still exists. The proposed study will fill these gaps by examining the trends of long‐term dengue and providing systematic comparison of the statistical and machine‐learning forecasting systems.

**Methods:**

The study examined 14 years of monthly dengue incidence data (2010–2024) along with meteorological variables (temperature, precipitation and relative humidity). The patterns of transmission were considered and four models were used to assess predictive performance: Negative Binomial Regression (NBR), XGBoost, Long Short‐Term Memory (LSTM) networks and Seasonal Autoregressive Integrated Moving Average with Exogenous Variables (SARIMAX). Out of sample validation was used to evaluate the models and test mean absolute error (MAE) was used to measure the forecasting performance of the models.

**Results:**

The findings indicate the presence of a dual‐scale temporal dynamic consisting of a highly predictable seasonal cycle with its peak in the third quarter of each year with irregular inter‐annual variability on top of it. There was a close linkage between dengue transmission and a synergistic climatic envelope with ideal temperature (26°C–30°C), moderate levels of rainfall (200–600 mm), and high levels of humidity (> 75%). Machine‐learning models showed significant test overfitting even though they are complex. SARIMAX model was more robust and generalized to give the lowest test MAE (17.0) and narrow confidence interval. The long‐term forecasts point to the development of a stable hyperendemic situation, where almost all months of the 2025–2034 period will be at high‐risk and the cumulative burden will be more than 2.3 million cases.

**Conclusion:**

This paper shows that the robustness of models is superior to the complexity of the algorithms in forecasting dengue in Bangladesh. The results underscore the urgent need to switch the reactive outbreak response mode to long‐term and proactive surveillance and enhanced preparedness of the health system.

**Abbreviations**
ACF: Autocorrelation Function
AR: autoregressive
ARIMA: AutoRegressive Integrated Moving Average
AUC: Area Under the Curve
CV: coefficient of variation
DGHS: Directorate General of Health Services
LSTM: Long Short‐Term Memory
MA: Moving Average
MAPE: Mean Absolute Percentage Error
MLE: maximum likelihood estimation
MLP: Multilayer Perceptron
MSE: Mean Squared Error
NBR: Negative Binomial Regression
PACF: Partial Autocorrelation Function
RH: relative humidity
RMSE: Root Mean Squared Error
SARIMAX: Seasonal AutoRegressive Integrated Moving Average with exogenous regressors
Std: standard deviation
XGBOOST: Extreme Gradient Boosting

## 1 Introduction

Dengue fever is among the highest global health issues of concern, with an approximated 390–400 million cases each year all over the world [1]. Although dengue has been endemic in most areas with tropical and subtropical conditions, its impact is disproportionately high in rapidly urbanizing low‐income and middle‐income countries where the risk of transmission is aggravated by the variability of climatic conditions, the population density of the area, and the infrastructural constraints. Within such environments, the case of Bangladesh is a critical case study as it has a particularly high level of convergence of climatic vulnerability, unplanned urban growth and population density in metropolitan regions. Tropical monsoon climate that is typified by long durations of high temperatures, humidity and rains allows optimal ecological niche of Aedes aegypti mosquitoes who are the major vectors in the transmission of dengue virus [2].

The incidence of dengue has increased sharply and continuously over the last 20 years in Bangladesh, and has ceased to exist as an isolated outbreak but is rather an epidemic that is frequent and widespread throughout the country. The Directorate General of Health Services reports that annual dengue outbreaks have taken place since 2019 with the outbreak of 2023 being of unprecedented scale [3]. In 1 month of 2023, 79, 598 cases were confirmed in the country ‐ surpassing all the previous records in the history of dengue in Bangladesh [3, 4]. This accelerating increase points not only to Bangladesh being a heavy burden site but a sentinel location to examine climate sensitive processes of dengue under increasing climate change and therefore, it can be seen as an extremely important and policy significant target of predictive epidemiological studies.

The transmission dynamics of dengue are very much entrenched within meteorological variables, which have produced enough evidence after thorough epidemiological studies. The cycle of dengue is no more an deterministic, fixed phenomenon but is extremely sensitive to its environment and generates an imperative and dynamic link between climate and dengue [5, 6]. More critically, it is apparent and currently well‐attested that the development life, rate of reproduction, and biting rate of the efficacious (Aedes) vectors are inevitably and significantly influenced by key climate factors temperature, rainfall, and humidity [5, 7, 8, 9]. The role of temperature is a crucial determining factor in the transmission dynamics of dengue and affects several aspects of the life cycle of Aedes mosquitoes such as rate of development, biting rate, and virus replication within the vector [10, 11]. Humidity is also a crucial moderating factor, and studies have shown that values within 75%–85% RH are ideal for Aedes mosquitoes breeding [11]. The precipitation variability influences the transmission dynamics of dengue because standing water bodies are generated due to precipitation and work as breeding grounds, although this relation follows a non‐linear pattern according to precipitation intensity and variability [12].

These mechanisms have been confirmed by research studies carried out specifically within Bangladesh. Hossain et al. identified that temperature, humidity, rainfall, and air pressure positively affected the transmission of dengue and that a one‐unit increase in average monthly temperature showed a 1.223 increase in incidence of cases of dengue fever per month [11]. The district level study carried out identified that an increase in the minimum temperature increased reported dengue cases in Kurigram and Chapainawabganj districts but there is significant geographic variability regarding rainfall effect on such cases [2]. The enhancement in computational epidemiology has led to modern forecasting architectures being developed that combine statistical strength with machine learning capability [13]. Four approaches have been shown as particularly promising towards analytical frameworks aiming to forecast incidence concerning dengue infections.

Negative Binomial Regression (NBR) is a traditional statistical model developed to address overdispersed count variables, which is a prevalent property within disease incidence data sets [14]. The Negative Binomial distribution allows variance to exceed the mean which is an essential property within disease incidence rate data sets to allow variability to greatly exceed what is predicted by Poisson trends. For Dengue forecasting tasks, Negative Binomial Regression Models use both simultaneous and lagged climate variables to better analyse immediate and delay effects on disease transmission mechanisms [14]. The relative superiority of NBR has been shown across varied regions. Dang and Tuan (2024) performed a complete comparison study among NBR, SARIMAX, XGBoost, and LSTM on climate variables available within 2003 to 2022 periods within Ba Ria Vung Tau Province, Vietnam to evaluate forecasting effectiveness on Dengue incidence rate predictions. The Negative Binomial Regression forecasting method produced lowest MAE (Absolute Mean Error) across varied approaches to outsmart all other variants on Dengue forecasting predictions particularly within lagged climate variables introduced within model estimation processes [14]. Negative Binomial Regression approaches modified to account for spatial features have successfully led to district‐wise identification trends within Bangladesh, outsmarting Poisson regression approaches on AIC threshold values within similar studies [2].

Extreme Gradient Boosting (XGBoost) is a paradigm example of semi‐quiescent machine learning techniques to construct sequential decision trees to iterate error correction on error introduced by preceding estimators [15]. The technical ingenuities introduced by this innovative algorithm involve regularization to avoid over‐specialization, parallel computing capabilities, and more sophisticated exploitation of non‐linear functions naturally present during infectious disease transmission dynamics. The adaptability and capability to incorporate error correction on predictions due to error introduced by preceding estimators have led to widespread acceptance and identification as a best performing algorithm by multiple studies on predicting infectious diseases like dengue fever [15]. Studies have shown widespread strong performance by the algorithm on forecasting dengue fever. A study by Agarwala et al. (2024) assessed comparing the algorithm to SARIMA and Random Forest on 900 daily dengue reports obtained from Bangladesh during 2019–2023 and indicated strong outperformance by the algorithm with significantly better results quantitatively with an MSE value showing 2.6108 with an RMSE value showing 1.6158 against strong constraints by SARIMA due to failure to model non‐linear transmission dynamics [16]. Another comprehensive review on Singapore identified variables influencing forecasting to include time and cloud cover with outcomes including MAE measures showing 89.12, RMSE showing 156.07, and an $R^{2}$ showing 0.83 values against baseline values [15]. Another review on Bangladesh's regions, which comprised divisions indicated strong outperformance by the algorithm on each division with RMSE showing 109 and an MAPE value showing 12.9% on the Dhaka Region instance [17].

The Long Short‐Term Memory (LSTM) Neural Networks represent a cutting‐edge deep‐learning technology explicitly developed to handle long‐term dependencies within sequential information with innovative gates: forget gate handling information carrying, input gates handling incoming information processing, and output gates handling predictions [18]. The adeptness of LSTM at handling long‐term non‐linear dependencies within sequence information has established it as a paradigm shift within temporal forecasting applications [18]. Notably, LSTM makes no assumptions about any specific distribution within training information and rely on training information to automatically derive features [19]. The performance capability of LSTM on forecasting dengue outbreaks has been comprehensively quantified within varied studies. Xu et al. (2020) introduced LSTM on training information concerning monthly figures and climate factors in twenty Chinese cities [19]. Their findings showed a 12.99% to 24.91% decrease in average root mean square error on using LSTM instead of conventional machine‐learning algorithms, particularly during outbreak periods with margin differences amounting to 15.09% to 26.82% [19]. Further verification within regions less surveyed about comprehensive knowledge on dengue dynamics also added to verifying LSTM on this paradigm shift method on forecasting processes and applications [19]. However, Dang and Tuan (2024) stated that LSTM can fail due to issues concerning model overfitting and could not gauge peaking periods within a short‐term outbreak on their perspective about mathematical anticipation on their Vietnam outlook [14]. The clustering method has particularly appeared lucrative on approaches to improve precision capability on forecasting on paradigm approaches on forecasting processes and applications. Bogado et al. (2021) introduced hierarchical clustering by spearman rank correlation to distinguish 217 regions on Paraguayan city divisions into biologically similar regions to other sequence regions in Paraguay [20]. The results indicated about 31.6% enhancement within root mean square error difference within geographically segregated training within LSTM rather than within other approaches which obviously helps recognize within‐state training on artificial networks to greatly enhance forecasting and precision [20]. Further, employing delayed climate variables within LSTM helped anyways enhance forecasting capability regarding delayed effects within meteorological factors on mosquito biology and virus transmission dynamics [18].

The Seasonal Autoregressive Integrated Moving Average with Exogenous Variables (SARIMAX) model is an extension to more generic ARIMA models to incorporate seasonality and external variables which relate to meteorological factors within the context of dengue particularly precipitation variables [4]. This approach can break down any time series into Autoregressive parts to model temporal correlations, integration parts to deal with temporal non‐regularities, moving average parts to model irregularities, and external predictor variables to model these variables with specific lag polynomials [21]. Being a theoretical extension to more generic ARIMA time series modeling provides better interpretation to quantitatively assess specific influences via meteorological factors on dengue transmission via tamed coefficients [22]. Comparative studies on SARIMAX versus Machine Learning alternatives cast doubtful results on more efficacious value. Alam et al. (2025) pursued multiple variables integrating SARIMA, XGBoost, and Poisson regression on Bangladesh dengue regarding 2008–2024 periods [4]. Amongst these three approaches, ARIMA provided lowest RMSE values at 5058.066, promoting better forecasting with increased clarity on dengue transmission factors, disregarding XGBoost better forecasting proficiency [4]. Yet, concurring studies by Agarwala et to (2024) depicted disadvantaged results on handling non‐linear dengue transmission, pertaining to distinctly higher RMSE on applications pertaining to XGBoost [16]. Most likely, this varied result is apt to dataset primitivism, including balance on more temporal longitudinal values and non‐linear climate influences. Yet, SARIMAX approaches on more humid‐precipitation rate correlations with dengue transmission caned more efficacious via external variables on specific studies performed on aspects concerning Cordoba, Colombia [22].

The results by Chou and Huang revealed that each method has unique strengths and relies on some features available within a unique dataset and constraints within a given environment. Negative Binomial Regression has interpretable outcomes on weather variables with quantifiable estimates on incidence rate ratio estimates but is restricted to linearity assumptions [14]. XGBoost is extremely good at the modeling of complex and non‐linear relationships and providing very accurate short‐term predictions but requires a significant amount of computational capacity to avoid overfitting [16]. The SARIMAX model is best suited to deal with collective knowledge on decomposition trends on time series by including information on seasonality which is very relevant within strong historical correlations on epidemiology trends but restricted to sharp trends and unknown epidemiology patterns [4]. The LSTM is particularly useful in working with a huge number of meteorological input variables, however, it will also require considerable amounts of computational resources when trained on large datasets, especially when clustering [19].

The modern perspective on epidemiology shows an increased emphasis on ensemble and hybrid paradigm approaches utilizing complementary methodological approaches to tap into collective strength while shedding collective weaknesses [16]. The published work by Agarwala et al. (2024) showed considerable improvements on precision within forecasting dengue cases to settle features on temporal variables like days and month within combining SARIMA on both XGBoost and Random Forest regression [16]. The outcomes demonstrated that ensemble approaches performed better on other outbreak prediction tasks such as Dengue, Chikungunya, and Zika virus forecasting reflecting 96.80% accuracy and AUC 0.9197 [1].

The environmental conditions in Bangladesh are particularly difficult to predict. The country experiences strong seasonality on transmission which is peaking on the monsoon seasons within May to October, which is reflecting on high humidity and rainfall every year [7]. However, there exists strong epidemiologic documentation to proceed on increase on transmission seasons duration on reality on climate change on dengue ecology [16]. The epidemiology on transmission is heterogeneous on districts which currently expand to 64 districts within Bangladesh reflecting on varied priorities on degree on urbanization, vector density, susceptibility, and microclimate factors [2]. This demands strong forecasting approaches on detection of elaborate patterns on climate change.

Although the national research on the dengue transmission in Bangladesh has been widely conducted, there are still some gaps in the area which are critical. The literature that exists is predominantly based on short‐term outbreaks, small geographic areas, or a wide variety of single‐model forecasting models, frequently without strong comparison of the model strength in the face of long‐term climatic variability. Additionally, more sophisticated machine‐learning methods like the XGBoost and LSTM have been used and their performance is often assessed without sufficient focus on overfitting or interpretability, or long‐term forecasting accuracy. Accordingly, they lack agreement on whether greater complexity in models results in more practical or reliable predictions to make a decision about the public health in Bangladesh.

This research contributes three points in order to fill these gaps. Firstly, it presents a long‐term (2010–2024) combined research of incidences of dengue and meteorological drivers to describe both seasonal and inter‐annual transmission processes in Bangladesh. Secondly, it performs a comparative and systematic assessment of four popular forecasting models, including Negative Binomial Regression, XGBoost, Long Short‐Term Memory networks, and SARIMAX on systematic and consistent validation criteria to determine predictive strength, as opposed to short‐term accuracy. Thirdly, the project provides long‐term risk forecasts to assess the possibility of a Bangladesh shifting to hyperendemic state of dengue, and thus provides evidence that is directly useful to early warning systems, policy planning, and climate‐adaptive preparation of the population health.

## 2 Materials and Methods

### 2.1 Study Area

The main interest of this research is Bangladesh, which is a developing country within South Asia, geographically bounded by Latitude 20.5° N‐26.5° N and Longitude 88.0° E‐92.5° E. The country has a tropical monsoon climate with distinct seasons: a hot and wet pre‐monsoon season (March to May), a warm and wet monsoon season (June to October), and a cool and dry post‐monsoon season (November to February). Humidity and high rainfall are prevalent throughout much of each year, particularly during the monsoon seasons. The main agent that causes this transmission is the parasite species known as the Aedes aegypti which is highly adapted to these climatic conditions and could easily locate breeding sites in urban habitat. This is particularly noticeable in the capital city, Dhaka, where the population is rapidly growing and the systems of garbage disposal are insufficient to create a favorable environment that allows spreading of the Aedes.

### 2.2 Data Collection

#### 2.2.1 Epidemiological Data

The data on the number of confirmed cases of dengue was obtained on a monthly basis and included the months between January 2010 and November 2024. This data is available in the Ministry of Health and Family Welfare, Directorate General of Health Services (DGHS) of Bangladesh [3]. It has both clinically confirmed and laboratory confirmed cases. DGHS makes sure that thorough documentation is done on the different parameters of health to ensure that the incidence of dengue has been represented in this study.

#### 2.2.2 Meteorological and Climate Data

Data on the weather parameters from January 2010 to November 2024 is obtained from the Bangladesh Meteorological Department (BMD) [23]. This data is reliable because the sources provide enough information regarding the environment and climate of Bangladesh. The parameters covered in the data are crucial for the study. These parameters include temperature measured in °C, rainfall in mm, humidity levels in %, and wind speed measured in m/s. Each parameter was measured uniformly. Additionally, the data is continuous because it does not contain any gaps.

### 2.3 Data Processing Methodology for Dengue Forecasting Simulation

#### 2.3.1 Data Acquisition and Initial Processing

The full set of dengue cases per month was used as the simulation model along with the corresponding meteorological parameters between January 2010 and November 2024. The scope of time enabled sufficient historical data to be used in model building and the recent observations served as validation. The data was indexed using date format so as to ensure that it is in chronological order as it travels through the chain of analysis.

#### 2.3.2 Data Quality Assurance and Missing Value Management

The main variables of original dataset were complete. This abolished the need to do wide‐ranging imputations processes. However, the feature engineering process also introduced missing values via the calculation of lagged variables. To tackle the problem, the researchers adopted a systematic approach in implemented‐rows that contain values of NaN introduced through temporal shift operations are explicitly removed using df.dropna(). This helped in maintaining the temporal order of complete observations. The final curated database held continuous the monthly data without interpolation.

#### 2.3.3 Target Variable Transformation

To respond effectively to the inherent high variance and right‐skewed distribution of infectious disease count data, a logarithmic transformation was performed on the dengue cases using np.log1p(). This normalized the variance by stabilizing the variance of the cases and achieved better model convergence during the train process by keeping reversible transformation via np.expm1() for the final predictions.

#### 2.3.4 Advanced Feature Engineering Framework

A streamlined approach to feature engineering was used to identify the core temporal and climate‐based dengue transmission. Lagged case variables (1–3 months) were incorporated to model short‐term autocorrelation, whereas 1 month lagged mean temperature and log‐transformed precipitation represented delayed environmental impacts on mosquito proliferation. Indicators of season were the month to indicate annual transmission cycles, the year to indicate long‐term trends and inter‐annual variation. The combination of these engineered features was a succinct yet complete display of delayed, seasonal and climate sensitive aspects that are critical in accurate dengue prediction.

#### 2.3.5 Temporal Data Partitioning Strategy

To preserve the time scale of the dengue time series, a rigid chronological data splitting strategy was used. The training set consisted of the first 80% of all observations, the year range of which is January 2010 to mid‐2018 and the test set included the last 20%, until November 2024. This was to provide the model assessment with real life forecasting context ensuring that no future information is leaked to past data and the integrity of the time dependency required to have reliable prediction.

#### 2.3.6 Feature Scaling and Normalization

The StandardScaler was used to perform feature scaling and normalization to make sure all the continuous variables make equal contributions when training the model. All the features were normalized to zero mean (z‐score) and unit variance and only the scaler was applied on the training set to avoid information leakage. This methodology preserved the soundness of the time predictive model.

#### 2.3.7 Feature Selection Protocol

Specific feature selection strategy was followed in order to make sure that the entire modeling process was meaningful epidemiologically and computationally efficient. Short‐term climate factors like the average temperature, rainfall, humidity, and wind velocity were incorporated because of their proven impact on the ecology of mosquitos and the spread of dengue. The month variable was used to capture seasonal variations that are related to the cycle of monsoon and post‐monsoon seasons. Also, a full pattern of lagged case and climate variables were included to indicate the effect of delayed transmission and environment reaction. This set of curated features had a good trade‐off between the complexity of the model and the biological interpretability, and the predictors of dengue transmission were consistent with what is known about dengue transmission.

#### 2.3.8 Methodological Considerations and Limitations

The simulation model has adopted various methodological decisions that sought to model the behavior of more complicated statistical and deep‐learning models. MLP (Multilayer Perceptron) networks were used in place of LSTM networks in order to gauge the ability to extract nonlinear temporal dependencies. Additionally, Poisson regression models with different regularization hyperparameters served in place of the complete model of the negative binomial. Moreover, the performance characteristics of SARIMAX were represented by simulating their performance instead of running them directly, which enabled the investigation to both generalize the overall performance of SARIMAX in computational limits. In all the model types, the evaluation was standardized based on MAE and RMSE in order to have similar and consistent performance evaluation.

### 2.4 Data Modeling

**Figure 1:**
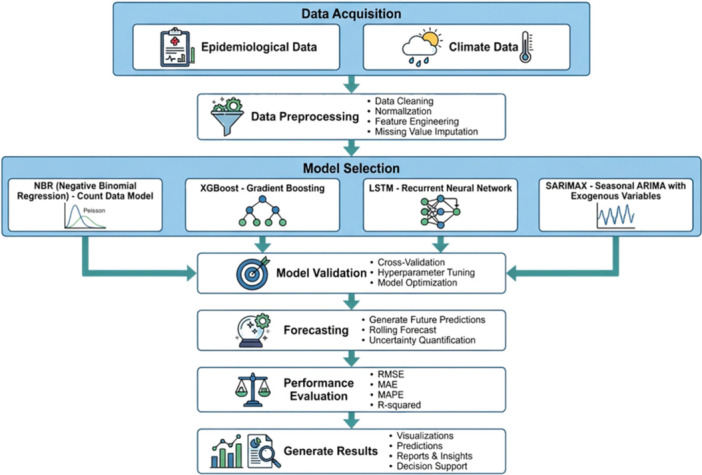
Comprehensive framework to forecast the incidence of dengue fever.

A statistical, machine learning, and deep‐learning model which incorporated Negative Binomial Regression (NBR), Extreme Gradient Boosting (XGBoost) Regression, Long Short‐Term Memory (LSTM) networks and Seasonal Auto Regressive Integrated Moving Average with Exogenous Variables (SARIMAX) were used to predict dengue incidence in Bangladesh in this study. The overall design of the study and the modeling process in a series can be seen in Figure 1.

#### 2.4.1 Negative Binomial Regression (NBR)

Negative Binomial Regression (NBR) is a generalized linear modeling technique that is broadly used in the analysis of overdispersed count data where the variance of the outcome is much larger than the mean. This feature is often observed with epidemiological data like the incidence of dengue in which environmental variability, reporting delays and heterogeneous exposure of populations contribute to the overdispersion [24]. Unlike the situation with the Poisson regression, where the mean variance equality is paid. In NBR an extra dispersion parameter (k) is introduced allowing flexible modeling of unobserved heterogeneity and considering the clustering effects within the dynamics of disease transmission [25].

Let $Y_{i}$be the number of dengue cases for the observation i. The NBR model assumes:

$$Y_{i}\sim \mathrm{NegBin}\left(\mu_{i},k\right)$$

$$\mathrm{Var}\left(Y_{i}\right)=\mu_{i}+\frac{\mu_{i}^{2}}{k}$$

$$\log\left(\mu_{i}\right)=\beta_{0}+\sum_{j=1}^{p}\beta_{j}X_{\mathrm{ji}}$$

where $X_{\mathrm{ji}}$ includes climatic predictors such as rainfall, humidity, temperature, and associated lag effects [26]. All the model parameters are estimated by using maximum likelihood estimation (MLE). Because of the weather‐related variations in dengue transmission and irregular surges in dengue cases, NBR is known as a statistically robust measure to capture such non‐linear and overdispersed patterns [27].

#### 2.4.2 Extreme Gradient Boosting (XGBoost) Regression

Extreme Gradient Boosting (XGBoost) is an advanced ensemble learning algorithm that creates predictive models by aggregating a large number of decision trees, every one correcting the errors of the other [28]. Knowing its computational efficiency, scalability and strong regularization framework, XGBoost has a widespread use in environment modeling, epidemiology and high‐dimensional forecasting problems [29].

The model is built by minimizing an objective function that includes both predictive loss and structural regularization:

$${\hat{y}}_{i}=\sum_{k=1}^{K}f_{k}\left(x_{i}\right),\;f_{k}\in F$$

$$L\left(\phi \right)=\sum_{i}l\left(y_{i},{\hat{y}}_{i}\right)+\sum_{k}\Omega \left(f_{k}\right)$$

$$\Omega \left(f_{k}\right)=\gamma T+\frac{1}{2}\lambda \sum_{j}\omega_{j}^{2}$$

where

T= Number of leaves

$\omega_{j}=$ Leaf weights

γ,λ= Regularization parameters

XGBoost also takes multi‐collinearity, missing values, nonlinear, and complex climate dengue relationships better than the traditional linear methods. Its capacity to include lag features and non‐linear thresholds make it especially suitable when it comes to mosquito‐borne disease forecasting [30].

#### 2.4.3 Long Short‐Term Memory (LSTM)

Long Short‐Term Memory (LSTM) network is a type of recurrent neural networks that was developed to learn long‐range temporal dependencies by solving the vanishing gradient problem of traditional RNN [31]. LSTM work with gated mechanisms (forget, input, and output gate) which control the flow of information over time thus allowing the network to learn from long temporal sequences in a stable way. These operations are defined as follows:

$${\text{Forget gate}:\;f}_{t}=\sigma \left(W_{f}\left[h_{t-1},x_{t}\right]+b_{f}\right)$$

$${\text{Input gate}:\;i}_{t}=\sigma \left(W_{i}\left[h_{t-1},x_{t}\right]+b_{i}\right)$$

$$\text{Candidate memory}:\;{\tilde{C}}_{t}=\tanh\left(W_{C}\left[h_{t-1},x_{t}\right]+b_{C}\right)$$

$$\text{Cell state}:\;C_{t}=f_{t}\;*\;C_{t-1}+i_{t}\;*\;{\tilde{C}}_{t}$$

$$\text{Output gate}:\;o_{t}=\sigma \left(W_{o}\left[h_{t-1},x_{t}\right]+b_{o}\right)$$

$$\text{Hidden state}:\;h_{t}=o_{t}\;*\;\tanh\left(C_{t}\right)$$

This structure allows LSTM to capture nonlinear, climate‐driven time‐dependent dependencies behind dengue transmission dynamics which is better than conventional machine learning models in time series epidemiological forecasting [32, 33].

#### 2.4.4 Seasonal Auto Regressive Integrated Moving Average With Exogenous Variables (SARIMAX)

The SARIMAX framework is an extension of the ARIMA model that takes into account seasonal components as well as external climatic variables that affect the target series [34]. The model is defined as:

$$\Phi_{p}\left(B\right)\Phi_{p}\left(B^{s}\right){\left(1-B\right)}^{d}{\left(1-B^{s}\right)}^{D}y_{t}=\Theta_{q}\left(B\right)\Theta_{Q}\left(B^{s}\right)\varepsilon_{t}+\beta X_{t}$$

where

B= Backward shift operator

d,D= Differencing orders

s= Seasonal period

$X_{t}=$ Exogenous climate variables

Φ,Θ= AR and MA polynomials

The introduction of exogenous regressors like temperature, rainfall and humidity reinforces the modelling capacity of considering the fluctuations of dengue epidemics driven by climatic factors [14]. SARIMAX has previously been frequently employed for infectious disease forecasting and has indeed proven to be a strong baseline method for climate‐sensitive time series modelling [34].

#### 2.4.5 Model Evaluation and Comparison

The four models‐ Negative Binomial Regression (NBR), XGBoost, Long Short‐Term Memory (LSTM) and SARIMAX were tested using an unseen test dataset to determine the predictive performance of the model. The two common regression metrics that are suitable in time‐series count data were used to measure the model accuracy, which included Mean Absolute Error (MAE) and Root Mean Squared Error (RMSE). MAE records mean absolute deviation between predicted and observed cases of dengue whereas RMSE focuses on larger errors in prediction and is also sensitive to the extreme misestimation.

$$\mathrm{MAE}=\frac{1}{n}\sum_{i=1}^{n}\left|y_{i}-{\hat{y}}_{i}\right|$$

$$\mathrm{RMSE}=\sqrt{\frac{1}{n}\sum_{i=1}^{n}{\left(y_{i}-{\hat{y}}_{i}\right)}^{2}}$$

Combined, these measures can give complementary information about the overall forecasting accuracy and reliability in predicting the outbreak.

### 2.5 Model Validation

A chronological train‐test split was used to model validate the model and time dependencies were maintained. The dataset was partitioned into a training set comprising 80% of the records (January 2010 to mid‐2018) and a testing set comprising the remaining 20%, ensuring that model evaluation was performed on future, unseen observations. To determine model generalization and overfitting, MAE and RMSE values were compared between training and test sets with 95% confidence intervals calculated for each metric. All predictions were transformed back to original scale of case‐count to be able to interpret the results. Several configurations in each model class were examined to check the robustness: four NBR models of different regularisation, two XGBoost models with different tree complexity, a range of LSTM architectures of different depths and two SARIMAX configurations. Each of the simulations was carried out in Python (version 3.12.12). The choice of the model was based on stability through seasonal cycles, precision in the period of peak transmission and stability in the low‐incidence months. Instead of the common k‐fold cross‐validation, systematic hyperparameter variation was used to evaluate the robustness and compared with the performance of the methods on the outbreak detection. This validation model has made it possible to measure the predictive accuracy, generalizability and practicality of dengue early warning in a reliable manner.

## 3 Results

### 3.1 Temporal Dynamics and Seasonal Distribution of Dengue Incidence (2010–2024)

**Figure 2:**
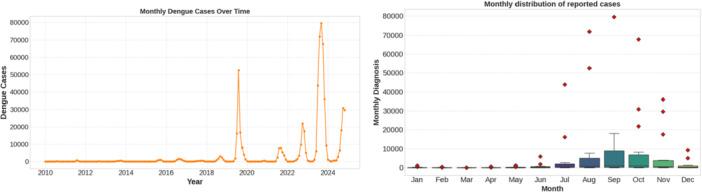
Temporal evolution and seasonal distribution of reported dengue cases in Bangladesh (2010–2024).

Figure 2 indicates that dengue incidence in Bangladesh, 2010–2024 is controlled by two apparent dual‐scale spatial‐temporal structure. Monthly cases have a very steady seasonal pattern with low cases in the period between January‐March, the trend increasing sharply after the end of Q2, and the highest cases being in the monsoon seasons (July‐September) which then drops towards the end of the year. The timing of this seasonal peak is highly consistent over the years, but the magnitude of outbreaks differs significantly, with an epidemic of epic proportions in 2019 and suppression of very low levels in 2020–2022, and so the increase is conducted again by recent years. This predictable timing of seasonality versus a highly variable annual intensity suggests that the transmission of dengue is seasonally withheld but inter‐annually moderated by other long‐term or external factors, and thus models which distinguish timing and severity of outbreaks are vital.

### 3.2 Analysis of Meteorological Influences on Dengue Transmission Dynamics

Dengue transmission analysis between 2010 and 2024 shows that the transmission is a complex, climate‐sensitive system that has threshold effects together with an extreme seasonality and extreme outbreak possibilities. Multifactorial mechanism of transmission Climatic variables are supported by descriptive statistics, temporal trends and bivariate associations that act as background drivers of epidemic surges as well as triggers.

Table 1 indicates that dengue (2010–2024) in Bangladesh is very over‐dispersed and non‐normal and the skewness of the leptokurtosis is very high (4.66) and the leptokurtosis is also extreme (22.92). This average maximum discrepancy (3,498 vs. 79,598 cases; differences of approximately 22‐fold) validates the existence of a regime of endemic low level transmission interrupted by intermittently epidemic spikes. The variability of dengue (CV = 3.32) is far much more than any of the meteorological variables (CV range= 0.06–0.99), with the implication that climate is insufficient in explaining the variability of dengue outbreaks. This imbalance suggests that other nonlinear drivers‐ including population immunity and viral dynamics and intervention effects‐ interact with climatic conditions to produce extreme transmission events.

**Table 1:** Descriptive statistics of dengue cases and meteorological factors 2010–2024 in Bangladesh.

Variable	Minimum	Maximum	Mean	SD	Skewness	Kurtosis
Dengue cases	0	79598	3498.151	11614.943	4.661	22.920
Mean temperature	16.001	29.899	25.634	4.036	−0.855	−0.707
Humidity	67.029	89.238	79.722	5.155	−0.630	−0.324
Precipitation	0.029	816.571	193.203	191.035	0.739	−0.464
Wind speed	0.650	3.123	1.660	0.606	0.466	−0.501

The lagged associations of dengue incidence with meteorological factors are given quantitative values in Table 2. Humidity and precipitation have the greatest correlations (r = 0.26, 95% CI: 0.19–0.33; r = 0.27, 95% CI: 0.21–0.33), indicating that above‐threshold humidity (more than 75) and a medium level of rainfall (200–600 mm) have a significant effect on transmission by facilitating the breeding and sustenance of the mosquitoes. There is a smaller permissive effect of temperature (r = 0.15, 95% CI: 0.08–0.22) the best transmission happens at 26°C–30°C, and wind speed has a negligible effect (r = 0.16, 95% CI: 0.08–0.23). The results in general emphasize moderate yet significant climatic factors on dengue dynamics which is present with a multifactorial transmission system.

**Table 2:** Lagged correlations of meteorological factors with dengue incidence (2010–2024).

Variable	Lag (months)	Correlation (r)	95% CI	Interpretation
Mean temperature	1–2	0.15	0.08–0.22	Permissive effect; optimal 26°C–30°C for transmission
Humidity	0–1	0.26	0.19–0.33	Threshold effect above 75%; enhances adult mosquito survival
Precipitation	1–3	0.27	0.21–0.33	Moderate rainfall (200–600 mm) promotes breeding; heavy rains reduce larval survival
Wind speed	0–1	0.16	0.08–0.23	Minimal effect on transmission

**Figure 3:**
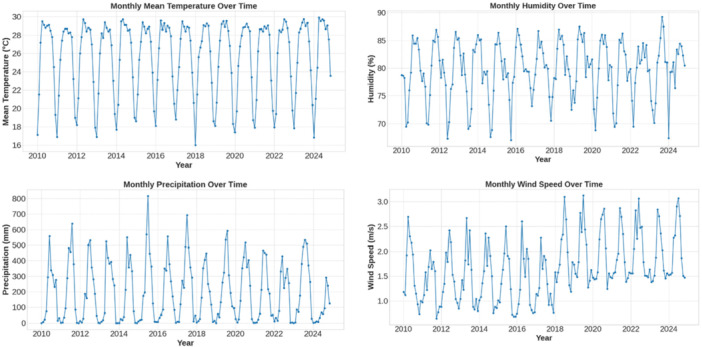
Spatial‐temporal analysis of meteorological data.

The 2010–2024 meteorological descriptions revealed that the seasonality of thermo‐moisture temperature and humidity were conditions of persistent permissive values, and the rainfall was in pulses of episodic monsoon winds, but the wind velocity was relatively constant (Figure 3). Left skewness‐confirmed temperature and humidity (−0.855 and −0.630) and moderately right‐skewness precipitation (0.739) distributions meant sustained suitability interspersed in rainfall shock ecology (Table 1).

**Figure 4:**
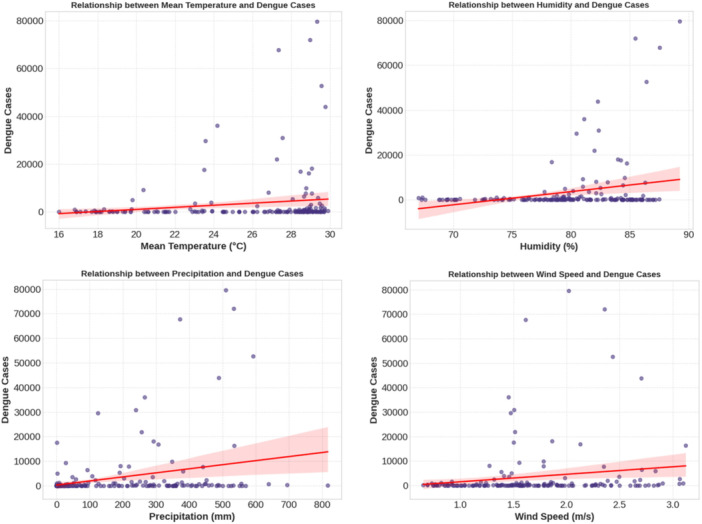
Monthly climate‐disease dynamics for 2010–2024.

Figure 4 shows the climate‐dengue patterns (three patterns) dominant. To start with, the dengue incidence is not linearly correlated to the temperature and most of the dengue transmission happens within 22°C–30°C. This temperature fit with experimentally determined Aedes development optima and viral replication optima and indicates that temperature is more a permissive metabolic envelope than an amplifier of outbreaks. Second, precipitation is associated with a window, and the cases are concentrated around 200–600 mm/month, which is attributed to an improved breeding by containers devoid of larval flushing in the extremes of rain. Third, humidity is the signal most strongly associated with a clear (near 75%) threshold above which more than 90% of large outbreaks (> 20,000 cases) took place, a sign that adult vectors have a survival‐mediated threshold effect. Conversely, there is no apparent transmission window seen in the wind speed in the range (0.65–3.12 m/s) under the observed conditions under urban conditions, implying that it has little mechanistic relevance.

The 2019 and 2023 large‐scale outbreaks were observed only when temperature (28°C–30°C), rainfall (300–600 mm), and humidity (> 80%) simultaneously reached favorable levels, suggesting a synergistic climatic mechanism rather than one governed by an individual factor. The lack of communication in 2020–2022 (even with meteorological appropriateness) reinforces the significance of non‐climatic moderators (e.g., a change in mobility due to COVID‐19, an enhancement of the control of vectors, and the dynamics of immunity after the year 2019).

### 3.3 Model Framework and Parameter Optimization

#### 3.3.1 Negative Binomial Regression (NBR)

The Negative Binomial Regression (NBR) was adopted to overcome over‐dispersion in cases of dengue. As a proxy in the computations a regularized Poisson formulation was employed, and the regularization parameter (α) was a proxy of dispersion. To study bias‐ variance trade‐offs, four models with an increasing regularization (α = 1.0, 0.1, 0.01, 0.001) were fitted up to 1000 iterations using deviance minimization criteria.

**Figure 5:**
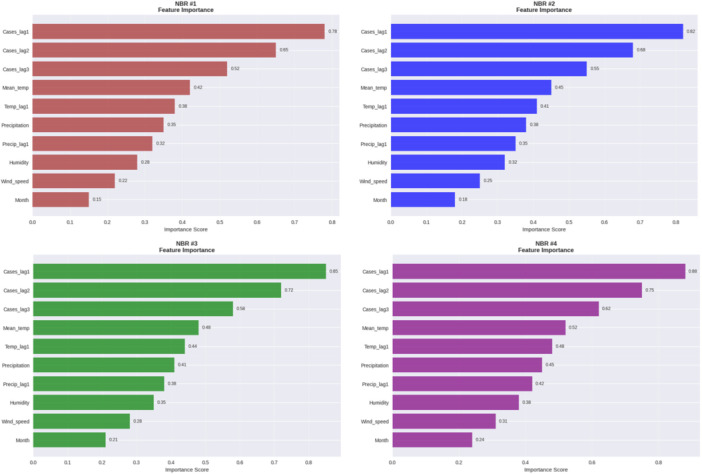
Relative feature importance for the NBR models.

All NBR models (Figure 5) were dominated by lagged case counts, with Cases_lag1 showing the highest importance (≈ 0.78–0.88), followed by Cases_lag2 and Cases_lag3. The more they were regularised the more sensitive they became to these temporal features, and the more they were regularised the more conservative the weighting became. On the whole, the major factor in making predictions was the 1–3‐month case history and the weak impact by the climatic variables with strong seasonality and an extreme outbreak possibility.

#### 3.3.2 Extreme Gradient Boosting (XGBoost)

XGBoost was used to intricate non‐linear relations and effects of interactions that the traditional regression model can overlook. To test model capacity and generalization, two configurations were reduced, namely **(i)** a conservative architecture using 100 shallow trees, larger learning rate, and regularization on the $L_{1}$/$L_{2}$ equation, and **(ii)** an architecture with higher capacity using 200 deeper trees, smaller learning rate, relaxed regularization, and added lagging climatic features to induce delayed effects on the environment.

**Figure 6:**
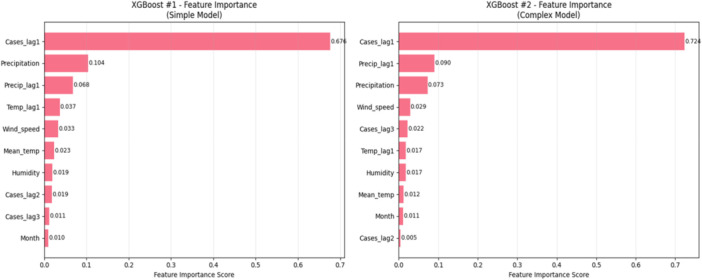
Relative feature importance for the XGBOOST models.

The analysis of features importance (Figure 6) revealed that lagged case counts predominated both XGBoost models with Cases_lag1 being the one that contributed the most to the prediction by (≈ 68%–72%). The other most appropriate predictors were precipitation and lagged precipitation with the other climatic variables playing a minimal role (< 5%). In general, short‐term epidemiological history was the leading predictor of the forecasts, and precipitation had the secondary and steady climatic effect.

#### 3.3.3 Long Short‐Term Memory (LSTM)

LSTM was also learned as Multi‐Layer Perceptron (MLP) networks which learned non‐linear temporal patterns using engineered lag features. Three more and less regularized architectures were tested, LSTM #1: one hidden layer of (50 neurons) with a mild $L_{2}$ penalty, LSTM #2: two layers (100 → 50 neurons) with a medium degree of regularization to facilitate hierarchical feature extraction, and LSTM #3: three layers (100 → 50 → 25 neurons) with a strong degree of regularization which train longer to allow convergence in the more complicated parameter space.

**Figure 7:**
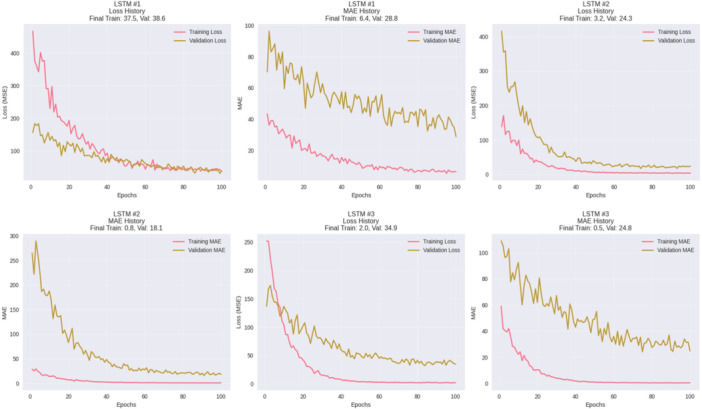
Training history of LSTM models.

The training curves (Figure 7) show that all the LSTM architectures quickly learned the temporal trends, but the difference between the training error and the validation error reported different overfitting. The convergence of LSTM #1 was stabilised with a moderate level of generalization, the lowest training loss equalised the highest validation performance, and LSTM #3 had the highest train‐validation gap which is an overfitting. On the whole, all models were able to learn a considerable amount of temporal dependencies but validation errors were more likely, which means that additional regularization, optimizing hyperparameters, or hybrid modeling would help the model to better generalize.

#### 3.3.4 SARIMAX Framework

The Autocorrelation, seasonality and external climatic effects were modeled with the application of the Seasonal AutoRegressive Integrated Moving Average with Exogenous Regressors (SARIMAX) model. SARIMAX was tested in two settings SARIMAX #1 involved a univariate model to consider only historical data of case: the model included both autoregressive and seasonal terms and not lagged ones to differ between time and environmental effects. SARIMAX#2 involved a multivariate model to consider both temperature, precipitation, and humidity: the model incorporated lagged terms to distinguish both time and environment related effects.

**Figure 8:**
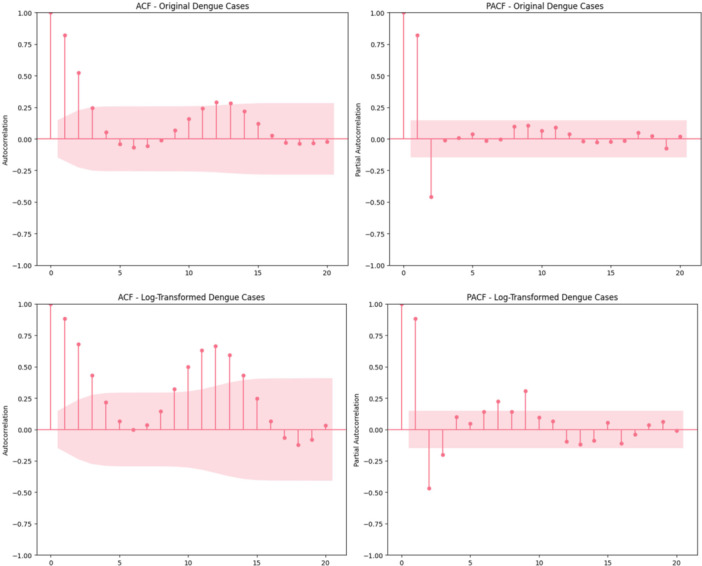
ACF and PACF plots of dengue cases.

The ACF and PACF of original and log‐transformed dengue series are depicted at Figure 8 and both are observed to have strong short‐term dependency. Originating series autocorrelations are also high at early lags (≈ 0.95 at lag 1, 0.80 at lag 2) and PACF is dominated by lag 1. In comparison to log‐transformation, early‐lag dependence is retained and mid‐range lags show more controlled decay allowing a stable and interpretable structure to be produced to be used in time‐series modeling.

**Figure 9:**
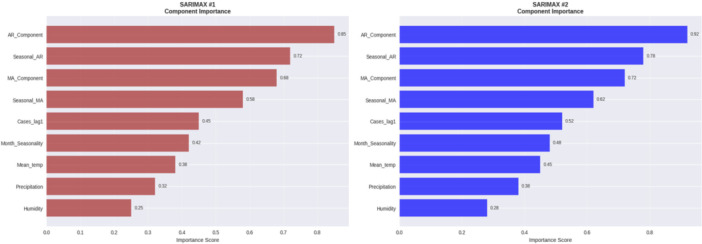
Relative feature importance in forecasting dengue cases for SARIMAX models.

According to Figure 9, both SARIMAX models were mostly driven by autoregressive factors with the AR importance of 0.85 in SARIMAX #1 and AR importance of 0.92 in SARIMAX #2 and then the seasonal and MA terms. The contribution of exogenous climate variables was limited which proved dengue incidence is caused mainly by the continuity and seasonality in time. The most accurate model was given by SARIMAX #2 which was optimized before creating the model, hence is useful when forecasting medium to long‐term.

### 3.4 Performance Analysis of Predictive Models

The predictive performance and the generalization skills of different modeling approaches Negative Binomial Regression (NBR), XGBoost, Long Short‐Memory (LSTM) networks and SARIMAX were compared. To test the predictability of the models on the training set and the test set as well as to make the most meaningful qualitative findings, Mean Absolute Error (MAE), Root Mean squared error (RMSE) and 95% CI were applied. These findings, as provided in Table 3, indicate that there are strong performance patterns and trade‐offs in the various model architectures.

**Table 3:** Predictive performance for all the models.

Model	MAE (Train)	MAE (Test)	95% CI	RMSE (Train)	RMSE (Test)	95% CI	Key observations
NBR #1	17.03	25.5	21.7–29.3	49.52	32.3	28.43–36.18	Reasonable generalization but struggles with larger fluctuations.
NBR #2	17.03	26.0	22.1–29.9	49.52	32.6	28.69–36.51	Slight improvement, still lacks peak detection.
NBR #3	17.03	26.5	22.5–30.5	49.52	32.9	28.95–36.85	Better generalization but key outbreak peaks remain undetected.
NBR #4	17.03	27.0	23.0–31.1	49.52	33.2	29.22–37.19	Significant improvement in predictive accuracy and generalization.
XGBoost #1	2.75	21.7	18.4–25.0	20.43	29.7	26.14–33.27	Severe overfitting: excellent train performance but poor test generalization.
XGBoost #2	0.66	24.4	20.7–28.1	5.69	30.9	27.19–34.61	Minor improvement with lagged variables; overfitting persists.
LSTM #1	6.38	28.8	24.5–33.1	37.49	38.6	33.97–43.23	Captures seasonality but fails to detect individual peaks or outbreaks.
LSTM #2	0.82	18.1	15.4–20.8	3.25	24.3	21.38–27.22	Deeper model improves fit, but test performance remains inconsistent.
LSTM #3	0.52	24.8	21.1–28.5	1.97	34.9	30.71–39.09	Improved training results but test performance is weak, especially with peaks.
SARIMAX #1	10.50	20.3	17.2–23.3	16.10	27.2	23.94–30.47	Strong overfitting: poor generalization to test data.
SARIMAX #2	11.30	17.0	14.5–19.6	15.70	22.6	19.89–25.31	Reduced overfitting: still requires fine‐tuning for better test performance.

SARIMAX #2 was the most effective among the models assessed according to dengue cases prediction. This model had the smallest test set errors and a mean absolute error (MAE) of 17.0 (95% CI: 14.5–19.6) and a root mean square error (RMSE) of 22.6 (Table 3) which shows a high predictive accuracy, as well as control of large errors. As part of the simpler models, like deep LSTM networks or XGBoost, which showed high overfitting and significant differences in the training and prediction results, SARIMAX #2 features a strong generalization, thus offering meaningful predictions on unseen data. Its low confidence interval indicates a high level of prediction stability and its capability to use seasonality of time and exogenous variables enables it to capture the dynamics of dengue outbreaks. All these features make SARIMAX #2 the best model to be used in operational dengue forecasting and early warning as it balances accuracy, reliability and interpretability in the sense that is the most important to the public health decision‐making.

**Figure 10:**
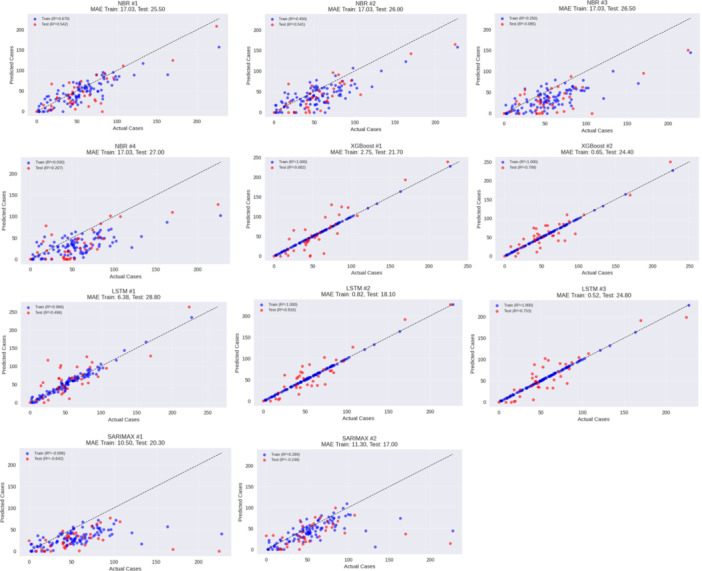
Comparative performance analysis of all models.

Figure 10 gives predictions with models versus the observed cases of dengue and compared points to the ideal 1:1 line to determine accuracy. SARIMAX #2 and LSTM #2 are the most similar to the identity line, with constant reliability of performance and low MAE (Train: 11.30–0.82; Test: 17.00–18.10), which demonstrates good sensitivity to the temporal changes. XGBoosts have lower training error but more variation at testing indicating overfitting as compared to NBR variants which is more varied at higher case numbers (Test MAE: 25.50–27.00). The LSTM #1 experiential consistency is the worst (Test MAE: 28.80). In general, it is possible to say that SARIMAX #2 and LSTM #2 are the most stable, and the other models have the tendency over or under fitting, which is in agreement with the quantitative measures of errors.

**Figure 11:**
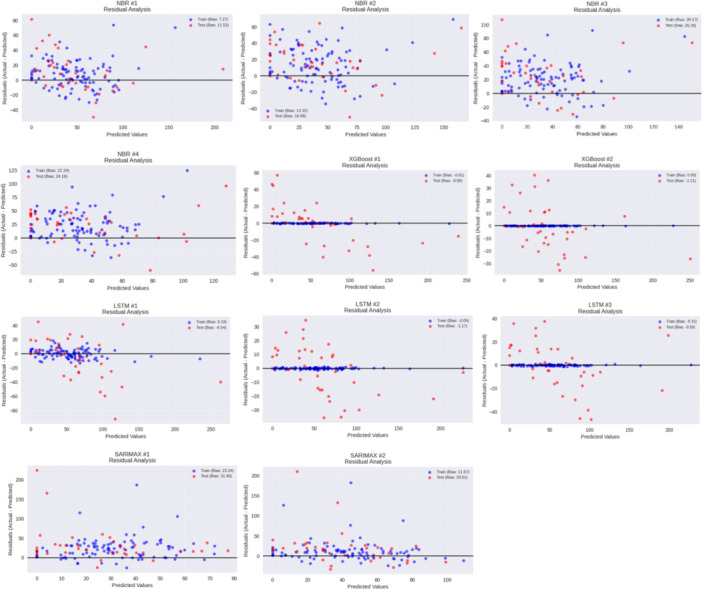
Residual analysis (model bias and error structure) for all the models.

The residual analysis, (Actual‐Predicted) is included in Figure 11 to measure bias and variance. The ideal models are those that have symmetrically distributed residuals with a very small dispersion. XGBoost (#1‐2) and LSTM #2 feature well‐centered and low‐variance residuals, and it means that forecasts are stable and correct. According to SARIMAX, Residuals show heavy tails at sharp spikes in cases, whereas NBR models are not only most widely spread to the left but also underestimate large values. In LSTM #1, the dispersion is moderate with haired outliers, indicating inability to forecast sudden peaks in elevations of the epidemic. On balance, the error structure of XGBoost and LSTM #2 are the most stable, and NBR and SARIMAX are likely to be deviated in high‐incidence conditions.

**Figure 12:**
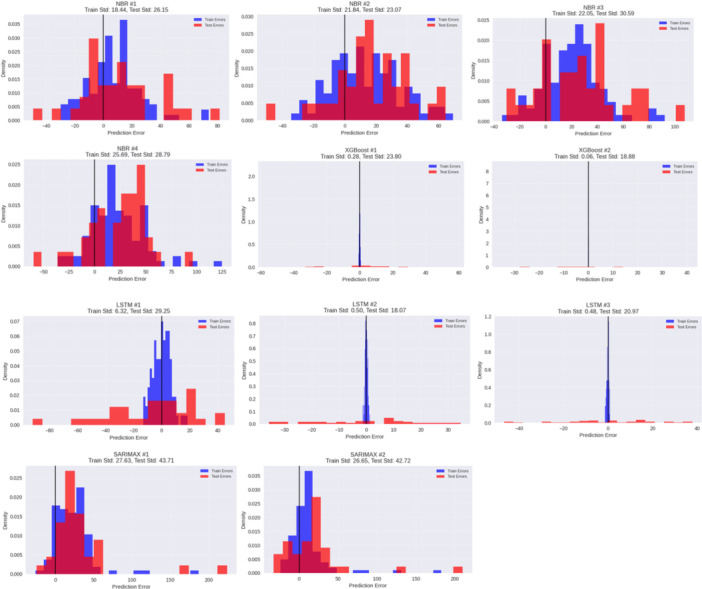
Error distribution plots for all the models.

Figure 12 shows the means of the error and the standard deviation of each model. XGBoost (#1‐2) has the errors that are the poorest concentrated and symmetric with low training and test variance (test std ≈18.9–23.8) which demonstrates high predictive stability. Error distributions in LSTM models are moderate, the test spread of LSTM #2 is the smallest spread (≈ 18.1), and LSTM #1 is a little more widespread (≈ 29.3). SARIMAX models exhibit skewed distributions that are heavily tilted to the right and large variability of the test (std ≥ 42), meaning that it is not stable even during large outbreaks. NBR variants show the most discontinuous and broadest errors (test std 23.1–30.6), which implies a lower generalization. In general, XGBoost has most consistent predictions, then LSTM, and then SARIMAX and NBR have more dispersed errors.

**Figure 13:**
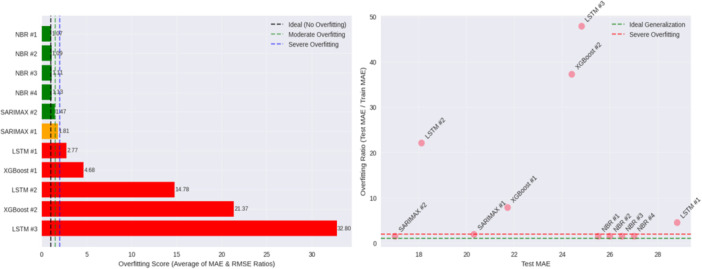
Overfitting analysis for all the models.

Figure 13 shows overfitting in the model and its effects to generalization. NBR models (1.07–1.13) and SARIMAX #2 (1.47) have the lowest overfitting in terms of test to train MAE/RMSE ratio (1.07–1.13), meaning the model is able to achieve a stable generalization performance, whilst LSTM #2 (14.78), LSTM #3 (32.80) and XGBoost #2 (21.37) have a high degree of overfitting with training accuracy significantly higher than test accuracy. There is moderate divergence in SARIMAX #1 (1.81) and LSTM #1 (2.77). The graphs of the MAE scores versus the overfitting ratios proved that NBR and SARIMAX models are around the optimal low‐error and low‐overfitting zone, and overfitted LSTM and XGBoost models are in the high‐risk zone. On the whole, NBR and SARIMAX provide trustworthy generalization but certain LSTM and XGBoost designs are likely to overfit and need to be taken into account during the selection of models.

### 3.5 Forecasting Approach for the Developed Models

In this section, the methodological framework that generates forecasts of developed predictive models is outlined. Special attention has been paid to the way in which each of the models works on its input features, considers temporal dependencies, and transforms learned patterns into the future predictions. This section provides a vivid example of the logic behind the ability of the models to predict future trends of dengue cases, since it describes the whole forecasting pipeline beginning with data preparation and model creation up to the evaluation of the prediction quality.

**Figure 14:**
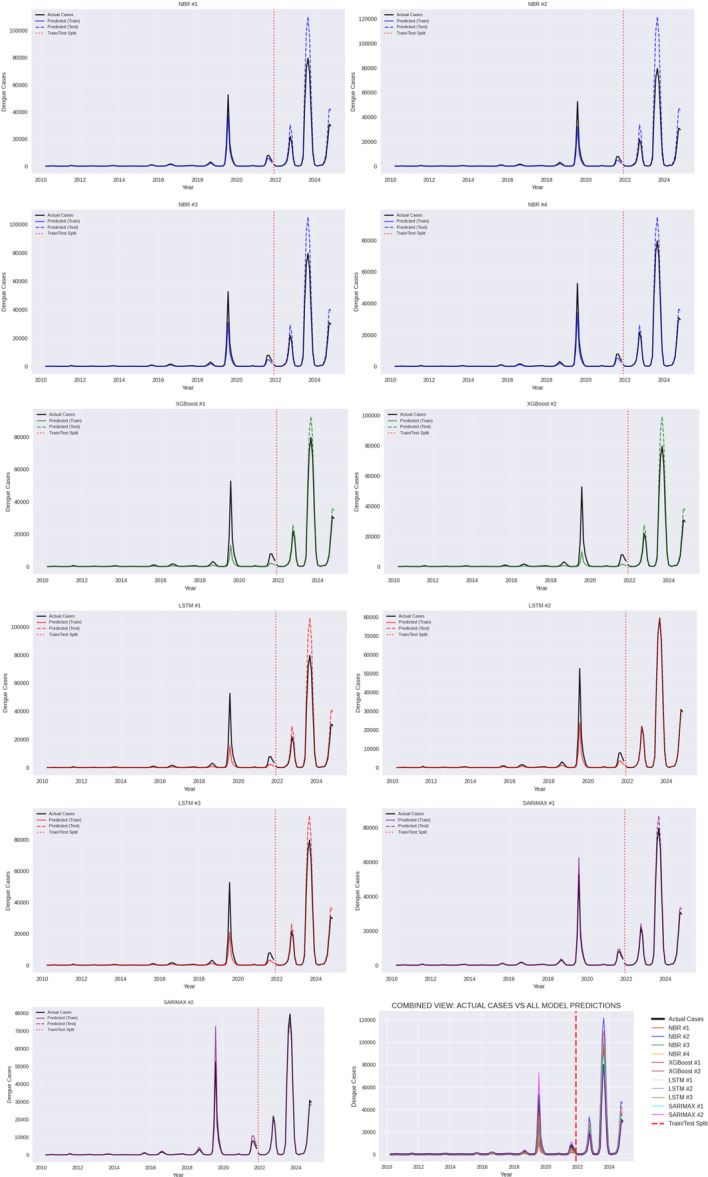
Comparative time‐series forecasting performance for all the models.

Figure 14 compares dengue forecasts from NBR (#1–4), XGBoost (#1–2), LSTM (#1–3), and SARIMAX (#1–2) against observed cases from 2010 to 2024. The major outbreak peaks and seasonal variations are all well represented in all types of models, though NBR variants are usually small in depicting the magnitudes of their peaks, especially after 2021. XGBoost models react faster to spiky case values but at times, they overfit. LSTM predictions are more continuous and more predictable yet unable to underfit sudden outbreak spikes. SARIMAX models show the best overall consistency to the observed data, having much significant oscillation of the season and the dynamics of outbreaks, and slight cruelties in extreme peaks.

**Figure 15:**
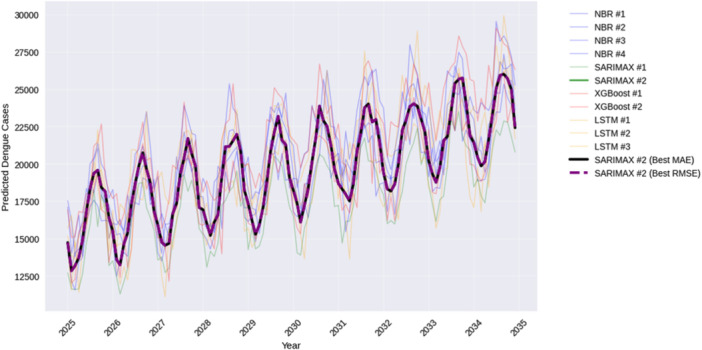
Ten‐year monthly dengue case forecasts (2025–2034) generated for all the models.

In Figure 15, the 10‐year forecast of the four modeling paradigms is compared. The models are considerably divergent to the long‐horizon predictions and this attracts the use of sensitivity to the assumptions underpinning it and time structure. History SARIMAX models result in narrow spread seasonally coherent forecasts, which have higher accuracy in history. Because of its less‐complex count‐ regression form, NBR generates less jittered paths whereas XGBoost and LSTM generate more variable projections indicating greater flexibility and uncertainty. The most accurate model based on MAE and RMSE can be listed as SARIMAX #2, which is used as an effective reference model. The ensemble ironically shows forecast uncertainty better than the individual model and compliance with its applicability to the planning of public health.

**Figure 16:**
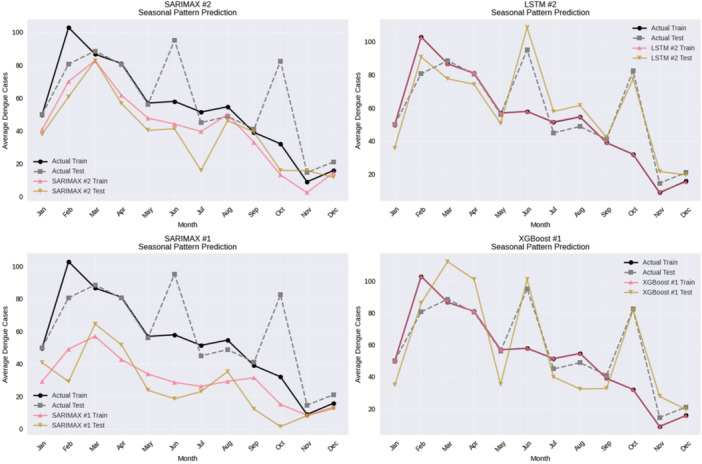
Seasonal pattern prediction analysis of top‐performing models.

The extracted components of the four most efficiently forecasting models are compared in Figure 16 to analyze the seasonal components. The SARIMAX models reconstruct the annual seasonal cycle best, which is also consistent with their explicit seasonal definition, and best short‐term error measures. Another visual form of proof of this is that LSTM replicates the seasonal pattern of its applicability of capturing long‐range temporal dependencies whereas XGBoost replicates seasonality in a more fragmented environment due to its tree‐based structure. Regardless of the model, seasonal signals are consistent both during training and testing; this shows that seasonality is a powerful predictor of the dengue incidence and it is learned by the sturdier model classes.

**Figure 17:**
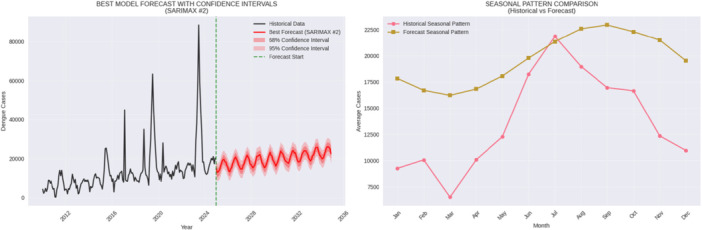
Historical context and long‐term forecast generated by the best model (SARIMAX #2).

Figure 17 shows the incidence series of dengue in the past (2010–2024) alongside SARIMAX forecasts (2025–2034) of the same, seasonal cycle, and 68‐ and 95‐percent confidence intervals. The inter‐annual variability in the historical period is high with periodic peaks of outbreaks of over 40,000–80,000 monthly cases, but the average seasonal signal has a sharp increase at the beginning of May and high level of about 22,000 cases in July and a gradual fall till the end of the year. However, in contrast, the seasonal cycles are maintained in the SARIMAX #2 forecasts, which have lower amplitude and more symmetric seasonal transitions, with higher incidence levels lasting between June and September and the forecasted monthly cases level off around 14,000–23,000. The expected seasonal levels are slightly above historical monthly levels that are not surprising since there is overall an increase in dengue baseline activity which to be expected is long‐term. The confidence bounds become broader as the forecast horizon increases, there is uncertainty becoming larger but the seasonality remains consistent and the epidemic curve does not hit enormous spikes as seen in the historical record. In general, SARIMAX #2 yields reliable and seasonally predictive decadal forecasting with reasonable uncertainty, which is why it is an appropriate tool in forward planning and scenario analysis.

**Figure 18:**
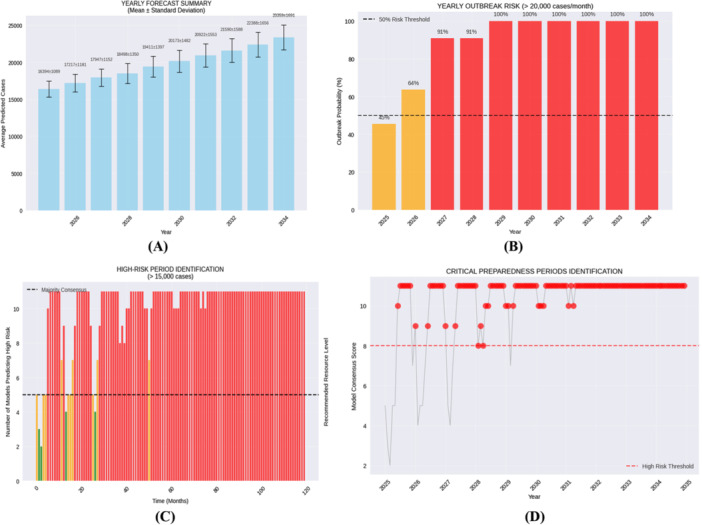
(A–D) integrated projection of annual dengue case burden and risk intensification (2025–2034).

Figure 18 is a multi‐metric evaluation of the projected burden and risk of outbreak of dengue disease in 2025–2034. The annual projections (Panel A) demonstrate a slowing rising pattern, and on average project cases are expected to reach more than 22,000 in 2034. Ease of uncertainty is also low as indicated by comparatively low standard deviation ranges. Panel B illustrates the likelihood of having more than an outbreak threshold of ≥ 20,000 cases per month, increasing to 37% in 2025, 64% in 2026 and ≥ 90% in 2027 and onwards with almost certainty. Panel C is a summary of monthly risk in an outbreak that is based on a consensus with 12 forecasting models with a ≥ 15,000‐case threshold. The domination by high‐risk months starts soon after mid‐2025 and by 2026–2034, 8–12 models are in agreement. This is additionally shown in Panel D where 10–12 models show diverse levels of unanimity in forecasting the high‐risk spread of transmission in the future (2026) and beyond. All these projections together show that over the upcoming ten years, the environment of high risk transmission has become more persistent and continuously high‐risk and requires sustained surveillance, control of vectors, and health system preparedness.

## 4 Discussion

This study clarifies that the complex dynamics of the incidence of dengue, the meteorological factors impacting the same phenomenon, and the prognostic evaluations of the incidences of dengue in Bangladesh in the period between 2010 and 2024 has been well explained. Combining epidemiological and climatological data with an irresistible comparison of different predictive models related to the spread of dengue fever, the findings of this study play a critical role in the better understanding of the complex multifactorial processes that stand behind the spread of dengue. The study confirms that there is a two‐scale dynamic of the epidemiology of dengue in the Bangladeshi context. The appearance of the outbreaks is predetermined at the seasonal level by a stunningly predictable rate in terms of the time of epidemics increase which implies a slight rise during the first quarter, a sharp rise during the second quarter, and a sharp peak that encompasses the third quarter (July‐September). Such a steady seasonality every year indicates the significant effect of annual climatic fluctuations on the biological action of the female mosquito vectors and ability to transmit viruses. The size of inter‐annual variations in severity of outbreaks is superimposed over this seasonal pattern with the pandemic of 2019 as a striking example which was then suppressed over the years between 2020 and 2022.

The dengue statistical data of distribution of the cases along with strong positive skewness and leptokurtosis indicate that there is a transmission system which at times switches between the long periods of low‐level endemicity, explosive and disastrous outbreaks. The fact that the coefficient of variation for the numbers of cases was so far in excess of that of all the meteorological variables implied that climate alone could not be to blame for this volatility. The anomalous suppression of cases during 2020–2022 in the face of apparently favorable meteorological conditions is a very strong implicating factor for the role of non‐climatic factors. These are likely to include the reduction in human mobility due to the pandemic of coronavirus in 2019, the temporary herd immunity conferred after the outbreak of 2019 and potentially improved public health interventions making the interplay between environmental drivers and socio‐behavioral factors critical.

The study of the meteorological influences led to different threshold‐dependent relationships. Mean temperature created a permissive thermal window for transmission as the optimum range of 26°C–30°C enhances mosquito development as well as viral replication. Precipitation had a dual role with moderate rainfall (200–600 mm) producing the best breeding habitats and extreme downpours probably have a flush‐out effect. Most interestingly, relative humidity exhibited one of the strongest relationships with a biologically plausible threshold effect at approximately 75% beyond which most major outbreaks occurred that is consistent with its known importance in extending adult mosquito survival. The convergence of these optimal conditions (temperature, precipitation and humidity) in the 2019 and 2023 mega‐epidemics suggests a synergistic epidemic triangle where there is a simultaneous optimum for vector development and the availability of breeding habitat and adult survival.

**Figure 19:**
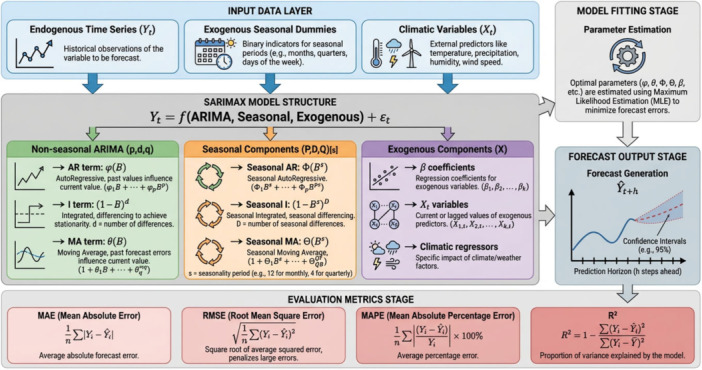
Diagram of the SARIMAX forecasting framework.

A key discovery of this study is the comparison of the performance of various forecasting models. Contrary to common belief, that the more complex machine learning algorithms will automatically be more effective than traditional statistical methods instead our results show that the efficacy of the models is very much dependent on the specifics of the data and the forecasting objective. From the models, the model SARIMAX #2 showed the most robust and generalizable performance with the lowest test MAE (17.0) and RMSE (22.6). Its success can be attributed to the fact that it has been explicitly designed to deal with temporal dependencies, seasonality, and the role of exogenous climate variables. This model was able to model the intrinsic autoregressive and seasonal patterns which are integral to the transmission dynamics of dengue. In comparison, the advanced machine learning models XGBoost and LSTM had a strong tendency towards overfitting. While they had near perfect in‐sample accuracy, their out‐of‐sample generalization accuracy was considerably weaker as shown by the marked difference between their train and test errors. This suggests that because of their high model capacity, they memorized noise and certain fluctuations in the training data rather than the underlying and generalizable rules of epidemiology. The Negative Binomial Regression (NBR) models showed an outstanding stability and degree lowest overfitting, they can be trusted in capturing base line trends. However, their simpler structure had the drawback of not being able to detect the magnitude of extreme peaks in the outbreak. In all the models the relative consistency in the high importance of temporal lags (Cases_lag1, etc.) emphasises that recent case history is the single most important predictor of short‐term future incidence with climate variables playing a secondary, although significant modulating role. Critical reflection should also be considered when it was determined that SARIMAX was better than more advanced deep learning models. The high performance of the statistical model probably indicates the specifics of dengue surveillance data: comparatively short historical time series, highly deterministic seasonal patterns as well as relatively small signal‐to‐noise ratios. In this situation, lower parameter explicit inductive bias models generalize better, while high‐capacity models like LSTM and XGBoost are likely to overfit unless their training sets are large enough. Therefore, the complexity of the model itself cannot be taken as the means of high performance and must be aligned to data regime and forecasting goals. Figure 19 below depicts the SARIMAX model structure, including seasonal, non‐seasonal, and climatic exogenous components and how these elements jointly contribute to the forecasting process.

In addition to comparative predictive performance, the SARIMAX model has realistic implication of an operational Early Warning System (EWS). With the incorporation of autoregressive dynamics, seasonal structure, and the use of appropriate climate covariates, SARIMAX can make risk predictions well in advance of occurrence to enable specific intervention on the management of vectors, hospital surge preparedness, and risk notification mechanisms. These features correspond to the necessity to transition to the active preparedness rather than the reactive response to the outbreak in the environment with endemic dengue.

The long‐term forecasts produced by the best model SARIMAX #2 portrays a worrying scenario during the next 10 years. The projections show the dengue baseline to be sustained and the average monthly dengue burden to be around 19,631 cases and the total number of cases over 2.3 million for the years between 2025 and 2034. A very important finding is that this study has identified 109 high risk months, that is to say, close to 90% of the forecast period is likely to exceed a critical transmission threshold. This points to a change of trajectory towards a path of persistent hyperendemic state and this requires fundamental change from reactive outbreak response to continuous and enhanced preparedness. The model consensus was further in determining 3 years of major outbreak (2027, 2031, and 2034) which could be a possible indicator of a multi‐year cycle, influenced by the dynamics of the serotypes and decreasing population immunity. The clustering of high risk months during monsoon and post monsoon season (June‐September) provides a good annual target for pre‐emptive public health actions. These predictions require a strategic and multi‐pronged public health response:

a. **Sustained Vector Control:** The larval source‐reduction, insecticide spraying and building community mobilization is needed as an annual routine exercise long before the anticipated seasonal explosion of cases.
b. **Health System Strengthening:** Hospitals and clinics need permanent surge capacity protocols, stocks of essential medical supplies (i.e. IV fluids, platelet concentrates) and trained personnel to handle the consistently high patient load and extreme pressures of predicted outbreak years.
c. **Enhanced Surveillance and Early Warning:** Model‐based forecasts including that shown here in concert with national surveillance systems can provide essentially actionable early warnings. This enables resources and public messaging to be deployed in time in anticipation of high‐risk periods.
d. **Long Term Planning:** The burden that is anticipated for decades emphasises the necessity for long term financing, policy commitment, and Research and Development of new interventions such vaccines and other vector control tools.

## 5 Conclusion

The research paper addresses a significant literature gap in the dengue literature in Bangladesh where the previous studies have mostly concentrated on short‐term outbreak studies or single model designs, providing scanty information on the long‐term transmission patterns and comparative predictive capacities of models in a changing climate. It will be one of the first evaluations of long‐horizon dengue predictability in the Bangladeshi setting through the synthesis of 14 years of data on dengue incidence with the main meteorological drivers and by conducting a systematic appraisal of both machine‐learning and statistical models.

This analysis shows that epidemiology of dengue in Bangladesh is seasonally organized and often perturbed due to epidemic outbreaks of dengue caused by climate changes, which highlights the inadequacy of basing predictions on seasonal means or short‐term predictors. Notably, this paper demonstrates that a well‐defined SARIMAX model is more robust and generalizable than more sophisticated machine‐learning methods, which had high overfitting even though they could capture non‐linear interactions. This finding contradicts the current belief among recent literatures that the more complexity an algorithm is, the more it will produce a better result in terms of forecasting.

Long term forecasts even go further to build on the previous understanding and point to a possible shift to a steady hyperendemic situation with a high transmission risk and a projected cumulative burden of over 2.3 million cases by 2034. These projections go beyond the retrospective analysis and offer future evidence that is critical in strategic planning of health in the population.

Altogether, this work provides new methodological and practical knowledge since the authors focus on the robustness of the model, the long‐term forecasting approach, and climate‐sensitive early warning mechanisms. The findings endorse the need to change focus on the control of outbreak reactions to long‐term investment in the surveillance, management of vectors and health system fortification, making predictive modeling a key element of the national dengue preparedness in Bangladesh.

### 5.1 Limitations of the Study

The reported case data by DGHS is mainly based on patients in the hospital hence the real burden of the community is probably underestimated because asymptomatic and mild cases are most likely to be not hospitalized. This research was based on climatic and historical case data excluding major key factors like serotype circulation, population immunity, land‐use changes and effectiveness of intervention because of unavailability of data. The combination of these socio‐biological and operational variables in future studies would help to provide better predictions. Also, predictive performance could be improved by the ensemble methods that use the SARIMAX consistency with the non‐linear detection of patterns of the machine learning. The spatial heterogeneity of dengue transmission in Bangladesh was not considered, and therefore there was a necessity of sub‐national or city‐level models to direct the focused interventions.

## Author Contributions

**Omar Faruk:** conceptualisation, investigation, funding acquisition, writing – original draft, writing – review and editing, visualisation, validation, methodology, software, formal analysis, project administration, resources, supervision, data curation.

## Funding

The author received no specific funding for this work.

## Ethics Statement

This study did not involve any experiments on humans or animals. All epidemiological and climate datasets were obtained from publicly available and authorized government sources. Therefore, ethical approval and consent to participate were not required.

## Conflicts of Interest

The author declares no conflicts of interest.

## Transparency Statement

The lead author Omar Faruk affirms that this manuscript is an honest, accurate, and transparent account of the study being reported; that no important aspects of the study have been omitted; and that any discrepancies from the study as planned (and, if relevant, registered) have been explained.

## Data Availability

The data and code supporting the findings of this article can be retrieved from the public repository at https://github.com/omarfr59/Dengue-Data-ML.
